# Application of IPSET-thrombosis in 1366 Patients Prospectively Followed From the Spanish Registry of Essential Thrombocythemia

**DOI:** 10.1097/HS9.0000000000000936

**Published:** 2023-07-18

**Authors:** Alberto Alvarez-Larrán, Beatriz Cuevas, Patricia Velez, Soledad Noya, Gonzalo Caballero-Navarro, Francisca Ferrer-Marín, Sara Carbonell, Manuel Pérez-Encinas, María Teresa Gómez-Casares, Raúl Pérez-López, Elena Magro, Ana Moretó, Irene Pastor-Galán, Anna Angona, María Isabel Mata-Vázquez, Lucía Guerrero-Fernández, José María Guerra, Gonzalo Carreño-Tarragona, Laura Fox, Ilda Murillo, Valentín García-Gutiérrez, Elvira Mora, Ruth Stuckey, Eduardo Arellano-Rodrigo, Juan Carlos Hernández-Boluda, Arturo Pereira

**Affiliations:** 1 Hospital Clínic Barcelona, Spain; 2 Hospital Universitario de Burgos, Spain; 3 Hospital de Mar, Barcelona, Spain; 4 Complexo Hospitalario Universitario de A Coruña, Spain; 5 Hospital Miguel Servet, Zaragoza, Spain; 6 Hospital Morales Messeguer, CIBERER, UCAM, IMIB, Murcia, Spain; 7 Hospital Clínico Universitario de Santiago de Compostela, Spain; 8 Hospital Doctor Negrín, Las Palmas de Gran Canaria, Spain; 9 Hospital Virgen de la Arrixaca, Murcia, Spain; 10 Hospital Príncipe de Asturias, Alcalá de Henares, Spain; 11 Hospital de Cruces, Barakaldo, Spain; 12 Hospital Clínico Universitario, Valencia, Spain; 13 Hospital Doctor Josep Trueta, Girona, Spain; 14 Hospital Costa del Sol, Marbella, Spain; 15 Complejo Asistencial de Palencia, Spain; 16 Hospital Son Llatzer, Palma de Mallorca, Spain; 17 Hospital Universitario 12 de octubre, Madrid, Spain; 18 Hospital Universitario Vall d’Hebron, Barcelona, Spain; 19 Hospital General San Jorge, Huesca, Spain; 20 Hospital Universitario Ramón y Cajal, IRYCIS, Madrid, Spain; 21 Hospital Universitario y Politécnico La Fe, Valencia, Spain

## Abstract

The International Prognostic Score of thrombosis in Essential Thrombocythemia (IPSET-thrombosis) and its revised version have been proposed to guide thrombosis prevention strategies. We evaluated both classifications to prognosticate thrombosis in 1366 contemporary essential thrombocythemia (ET) patients prospectively followed from the Spanish Registry of ET. The cumulative incidence of thrombosis at 10 years, taking death as a competing risk, was 11.4%. The risk of thrombosis was significantly higher in the high-risk IPSET-thrombosis and high-risk revised IPSET-thrombosis, but no differences were observed among the lower risk categories. Patients allocated in high-risk IPSET-thrombosis (subdistribution hazard ratios [SHR], 3.7 [95% confidence interval, CI, 1.6-8.7]) and high-risk revised IPSET-thrombosis (SHR, 3.2 [95% CI, 1.4-7.45]) showed an increased risk of arterial thrombosis, whereas both scoring systems failed to predict venous thrombosis. The incidence rate of thrombosis in intermediate risk revised IPSET-thrombosis (aged >60 years, *JAK2*-negative, and no history of thrombosis) was very low regardless of the treatment administered (0.9% and 0% per year with and without cytoreduction, respectively). Dynamic application of the revised IPSET-thrombosis showed a low rate of thrombosis when patients without history of prior thrombosis switched to a higher risk category after reaching 60 years of age. In conclusion, IPSET-thrombosis scores are useful for identifying patients at high risk of arterial thrombosis, whereas they fail to predict venous thrombosis. Controlled studies are needed to determine the appropriate treatment of ET patients assigned to the non-high-risk categories.

## INTRODUCTION

Essential thrombocythemia (ET) is a chronic myeloproliferative neoplasm (MPN) in which thrombosis prevention is the main goal of treatment.^1^ Based on the age and history of thrombosis, classic risk stratification established 2 risk categories, with high-risk patients being candidates for cytoreduction.^1–3^ The International Prognostic Score of thrombosis in Essential Thrombocythemia (IPSET-thrombosis) is a new prognostic system that incorporates the presence of cardiovascular risk factors (diabetes, arterial hypertension, and smoking) and the *JAK2*V617F mutation to the classical risk factors.^4^ IPSET-thrombosis stratification established 3 risk groups categorized into low, intermediate, and high risk with a corresponding annual rate of 1.19%, 2.26%, and 4.88% thrombotic events, respectively.^4^ A subsequent revision of the IPSET-thrombosis was made to further refine the prognostic stratification.^5^

The European LeukemiaNet recommends using the IPSET-thrombosis score to assess the risk of thrombosis, while the National Comprehensive Cancer Network recommends using the revised IPSET-thrombosis for decision-making on thrombosis prevention strategies.^1,6^ However, despite the potential usefulness of the IPSET-thrombosis scales, they have had limited application in routine clinical practice, mainly due to the lack of prospective studies confirming the original observations.^7,8^ In addition, because variables included in the IPSET-thrombosis and the revised IPSET-thrombosis are also associated with increased mortality,^9,10^ the question arises whether death unrelated to thrombosis may compete with the accurate prognostication of vascular events.

The present study has several objectives. First, to test the performance of the IPSET- and revised IPSET-thrombosis in predicting thrombosis in the context of death as a competing risk. Second, to investigate whether these prognostic models are equally effective in predicting thrombosis either in the arterial or venous territories. Third, to investigate whether there are patients who might not benefit from cytoreductive therapies to prevent thrombosis. To achieve these goals, we analyzed a large series of ET patients recruited to the Spanish Group of Ph-negative Myeloproliferative Neoplasms (GEMFIN) registry who were prospectively followed.

## PATIENTS AND METHODS

The Spanish Registry of Essential Thrombocythaemia is a nationwide, noninterventional prospective study started in 2015 by the GEMFIN registry. Patients diagnosed with ET according to the World Health Organization criteria after the year 2000 were eligible for inclusion. Informed consent for the scientific use of patients’ clinicohematologic data was obtained, and the study was approved by the local ethics committees. By June 2022, a total of 2682 patients were included in the registry, 1366 of them with prospective follow-up were selected for the present study. The remaining patients without prospective update were discarded.

The registry allows the inclusion of previously diagnosed and newly diagnosed patients. Clinical data at ET diagnosis and during follow-up including age, sex, systolic and diastolic blood pressure, cholesterol levels, smoking habit, medication for blood pressure, diabetes, therapy for ET, and complications were collected retrospectively in those patients included after diagnosis. After inclusion, patients were prospectively followed for therapy changes and complications. Data were entered by the attending physician or local investigators into an electronic case report form accessible at the scientific area of GEMFIN website.

The IPSET-thrombosis score was calculated at diagnosis as previously described.^4^ Patients were stratified into low-risk (0–1 points) intermediate-risk (2 points), and high-risk (≥3 points) categories. Patients were also stratified according to the revised IPSET-thrombosis as very low risk (aged ≤60 years, no thrombosis history, and *JAK2* unmutated), low risk (aged ≤60 years, no thrombosis history, and *JAK2* mutated), intermediate risk (aged >60 years, no thrombosis history, and *JAK2* unmutated), and high risk (thrombosis history or age >60 years and *JAK2* mutated).

The main outcome of the study was the occurrence of a first thrombotic event. Arterial thrombosis included coronary artery disease, stroke/transient ischemic attack, peripheral artery disease, and other arterial thromboses. Venous thromboembolic events (VTEs) included superficial thrombophlebitis, deep vein thrombosis, pulmonary thromboembolism, splanchnic vein thrombosis, and other vein thromboses.

We defined the period at risk of thrombosis as the time elapsed from the diagnosis of ET to the first thrombotic event, progression to a higher risk category, death, or last visit, whichever occurred first. We forcibly censored the survivors’ follow-up at 10 years from diagnosis to avoid predicting very late events. Exposition to antiplatelet agents, anticoagulants, or cytoreductive therapies was computed as the fraction of the period at risk that patients were on each of these treatments.

The cumulative incidence of thrombosis was calculated by taking death as a competing risk. Multivariate analyses of factors predicting thrombosis were done within the framework of competing risks by the method of Fine and Gray, in which the interpretation of subdistribution hazard ratios is similar to that of hazard ratios in the Cox model.

Incidence rates of thrombosis were calculated for the revised IPSET-thrombosis categories as assigned at diagnosis and after switching from very low- to intermediate-risk (dynamic intermediate-risk) and from low- to high-risk (dynamic high-risk category) because patients reached their 60th birthday without presenting thrombosis during the period at risk in the low-risk category. The effect of cytoreduction on the risk of thrombosis was estimated by calculating the incidence rate ratio of first thrombotic events, and it was expressed as the number of events per 100 patients-year. All the statistical analyses were performed with Stata, version 14 (www.stata.com).

## RESULTS

### Baseline characteristics, IPSET-thrombosis stratification, and therapy

Disease characteristics at diagnosis are shown in Table 1. Median time elapsed from ET diagnosis to inclusion in the registry was 3.9 years, with 373 patients included in the first year after ET diagnosis. Median age at diagnosis was 63 years, and 63% of patients were female. ET genotypes were *JAK2*V617F, *CALR*, *MPL*, and triple negative in 65%, 18%, 4%, and 12% of the patients, respectively. The distribution of patients according to the classical risk stratification, IPSET-thrombosis, and revised IPSET-thrombosis are shown in Table 2 and Suppl. Figures S1-S3.

**Table 1:** Main Clinical Characteristics at Diagnosis in 1366 Patients Included in the Spanish Registry of Essential Thrombocythemia

Age, Median (Range)	63 (10–95)
Female sex, n (%)	858 (63)
Bleeding before diagnosis, n (%)	40 (3)
Cardiovascular risk factors, n (%)	849 (62)
Diabetes, n (%)	215 (16)
Therapy for blood pressure, n (%)	707 (52)
Smoking, n (%)	186 (14)
Thrombosis before diagnosis, n (%)	192 (14)
Symptoms at diagnosis, n (%)	
Pruritus	97 (7)
Microvascular disturbances	216 (16)
Arterial thrombosis	49 (4)
Venous thrombosis	24 (2)
Bleeding	28 (2)
Hemoglobin, g/L, median (range)	142 (63–184)
Leukocyte count × 10^9^/L, median (range)	8.76 (4.1–30)
Platelet count × 10^9^/L, median (range)	700 (409–2700)
Genotype^*a*^, n (%)	
*JAK2V617F*	882 (65)
*CALR*	240 (18)
*MPL*	55 (4)
Triple negative	158 (12)

^*a*^Five additional cases not included in the table were double mutated JAK2V617F/MPL n = 4 and JAK2V617F/CALR n = 1. Full genotype was not available due to lack of MPL status in 26 *JAK2*V617F-negative cases.

**Table 2:** Distribution of Patients According to the Classical Risk Stratification, IPSET-thrombosis, and Revised IPSET-thrombosis in 1366 Patients From the Spanish Registry of Essential Thrombocythemia

Classical Risk Stratification	
Low	530 (39)
High	836 (61)
IPSET-thrombosis	
Low, n (%)	275 (20)
Intermediate, n (%)	343 (25)
High, n (%)	748 (55)
Revised IPSET-thrombosis	
Very low, n (%)	211 (15)
Low, n (%)	319 (23)
Intermediate, n (%)	207 (15)
High, n (%)	629 (46)

IPSET-thrombosis = International Prognostic Score of Thrombosis in Essential Thrombocythemia.

Overall, antiplatelet therapy was started in 1158 (85%) patients, cytoreduction in 1030 (75%) patients, and anticoagulation in 118 (9%) patients. The mean percentage of the follow-up time exposed to antiplatelets, cytoreduction, and anticoagulation was 76%, 62%, and 7%, respectively.

### Survival

After a median follow-up of 7.1 years from diagnosis (interquartile range [IQR], 3.7–11.9), 163 (12%) patients had died, and 139 had presented at least 1 episode of thrombosis. The IPSET-thrombosis, the revised IPSET-thrombosis, and the classic 2-tier prognostic model for thrombosis were strong predictors of mortality, both from any cause (Suppl. Figure S4) and after excluding patients with prior thrombosis (data not shown). Causes of death are provided in Suppl. Table S1.

### Performance of prognostic model for thrombosis

**Figure 1.**
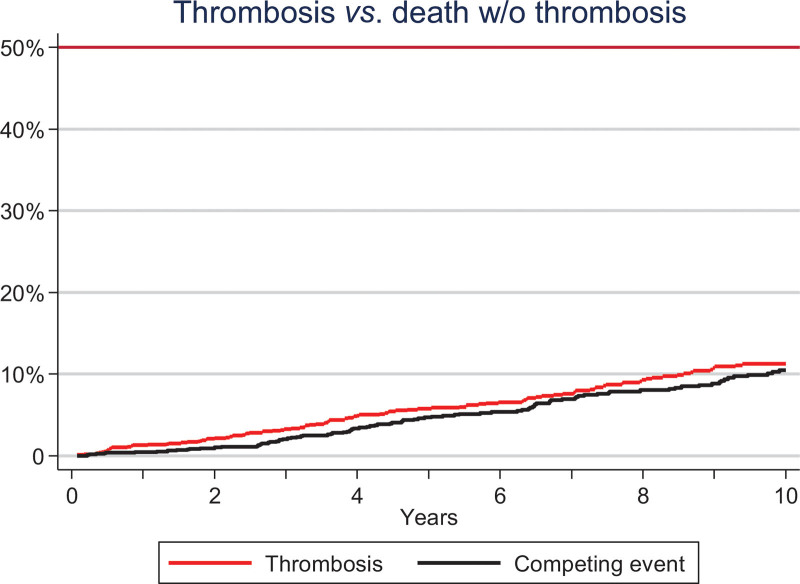
**Cumulative incidence of thrombosis taking death as a competing event in 1366 patients prospectively followed from the Spanish Registry of Essential Thrombocythemia.** Red line denotes thrombosis and black line death without thrombosis.

Time at risk for thrombosis, as defined in Material and Methods, amounted to 8521 patients-year with a median follow-up of 6.6 years (IQR, 3.4–10). During the period at risk, 112 had died without prior thrombosis, and 106 (7.8%) presented an episode of thrombosis (66 arterial; 40 venous). Types of thrombosis are provided in Suppl. Table S2. The cumulative incidence of thrombosis at 5 and 10 years, taking death as a competing risk, was 5.7% and 11.4%, respectively (Figure 1) and the annual rate was 1.2 thrombotic events per 100 person-year (0.77% and 0.46% per year for arterial and venous events, respectively).

**Figure 2.**
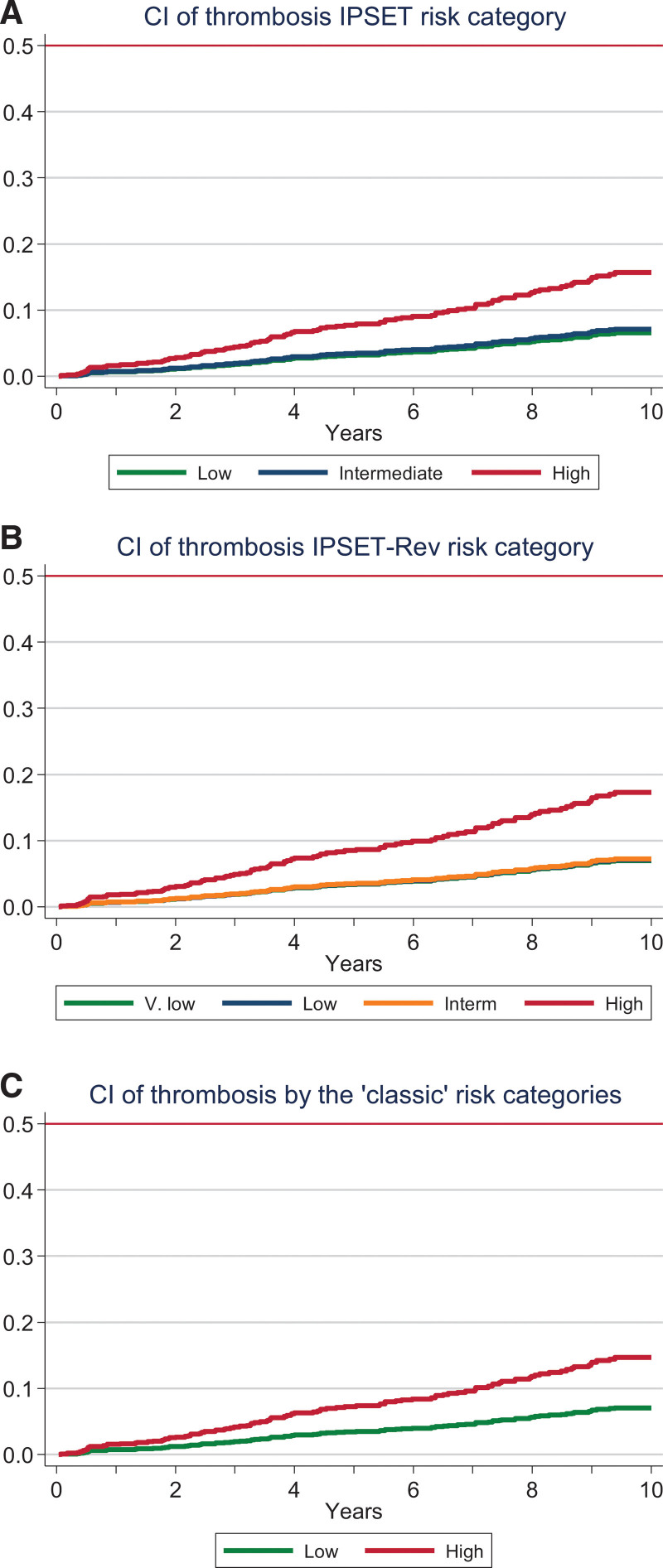
**CI of thrombosis according to the IPSET-thrombosis (A, IPSET), revised IPSET-thrombosis (B, IPSET-Rev), and classical risk stratification (C, classic) in 1366 patients prospectively followed from the Spanish Registry of Essential Thrombocythemia.** Patients were censored when they change to high-risk category or at 10 years after diagnosis. CI = cumulative incidence; IPSET-thrombosis = International Prognostic Score of Thrombosis in Essential Thrombocythemia.

Figure 2 shows the cumulative incidence of thrombosis according to the risk categories of IPSET-thrombosis, revised IPSET-thrombosis, and the classic 2-tier prognostic model. As shown in the Figure 2 and Table 3, a significantly elevated incidence of thrombosis was observed in the highest risk category of each prognostic model as compared with the remaining categories in the model (low for the classical, low and intermediate for the IPSET-thrombosis, and very low, low and intermediate for the revised IPSET-thrombosis). In contrast, these lower risk categories did not differ from each other in terms of thrombotic risk (Figure 2; Table 3). Adjustment for time on treatment with antiplatelet agents, cytoreductive drugs, or anticoagulants did not significantly modify these results (data not shown).

**Table 3:** Statistical Comparison of Risk Categories Within the IPSET-thrombosis, Revised IPSET-thrombosis, and the 2-tier Classical Prognostic Model for Thrombosis in ET

Risk Category	SHR (95% CI)	*P*-value
IPSET-thrombosis		0.002
Intermediate vs low	1.1 (0.5-2.2)	
High vs low	2.5 (1.4-4.5)	
High vs intermediate	2.3 (1.4-3.9)	
Revised IPSET-thrombosis		0.001
Low vs very low	1.0 (0.5-2.2)	
Intermediate vs very low	1.0 (0.4-2.4)	
Intermediate vs low	1.0 (0.5-2.2)	
High vs very low	2.6 (1.4-4.9)	
High vs low	2.5 (1.5-4.4)	
High vs intermediate	2.5 (1.3-4.8)	
Classical 2-tier model		0.005
High vs low	2.2 (1.4-3.3)	

SHR and the 95% CIs were estimated in the context of death as a competing risk for thrombosis.

CI = confidence interval; IPSET-thrombosis = International Prognostic Score of Thrombosis in Essential Thrombocythemia; SHR = subdistribution hazard ratios.

We further investigated the ability of IPSET-thrombosis, revised IPSET-thrombosis, and the classic 2-tier model to separately estimate the risk of venous or arterial thrombosis. As summarized in Table 4, the high-risk category of the 3 prognostic models was able to identify patients at increased risk of arterial thrombosis, but all 3 models failed to predict the risk of venous thrombosis.

**Table 4:** Rates and Risk of Arterial and Venous Thrombosis According to the IPSET-thrombosis, Revised-IPSET-thrombosis, and Classical Risk Stratification in 1366 Patients From the Spanish Registry of Essential Thrombocythemia

	Arterial Thrombosis				Venous Thrombosis			
	No. Events	10 y Probability	SHR (95%CI)	*P*-value	No Events	10 y Probability	SHR (95%CI)	*P*-value
IPSET-thrombosis				<0.001				0.8
Low, n = 275	6	3.3%	Ref.		7	3.9%	Ref.	
Intermediate, n = 343	7	3%	0.97 (0.3-2.9)		10	5%	1.2 (0.4-3.1)	
High, n = 748	53	12%	3.7 (1.6-8.7)		23	5%	1.35 (0.6-3.1)	
Revised IPSET-thrombosis				<0.001				0.5
Very low, n = 211	6	4.1%	Ref.		5	3.4%	Ref.	
Low, n=319	8	3.5%	0.93 (0.3-2.7)		8	3.7%	1.1 (0.4-3.4)	
Intermediate, n = 207	5	4%	0.95 (0.3-3.1)		5	5.4%	1.1 (0.3-3.9)	
High, n = 629	47	13%	3.2 (1.4-7.45)		22	6%	1.8 (0.7-4.6)	
Classical score				<0.001				0.2
Low, n = 530	14	3.8%	Ref.		13	3.6%	Ref.	
High, n = 836	52	10.8%	2.7 (1.5-4.9)		27	5.9%	1.5 (0.8-2.9)	

SHR in comparison with the reference group (lowest risk category in each model). Ref. denotes reference group for comparisons. *P*-value corresponded to global test for each model.

CI = confidence interval; IPSET-thrombosis = International Prognostic Score of Thrombosis in Essential Thrombocythemia; SHR = subdistribution hazard ratios.

**Figure 3.**
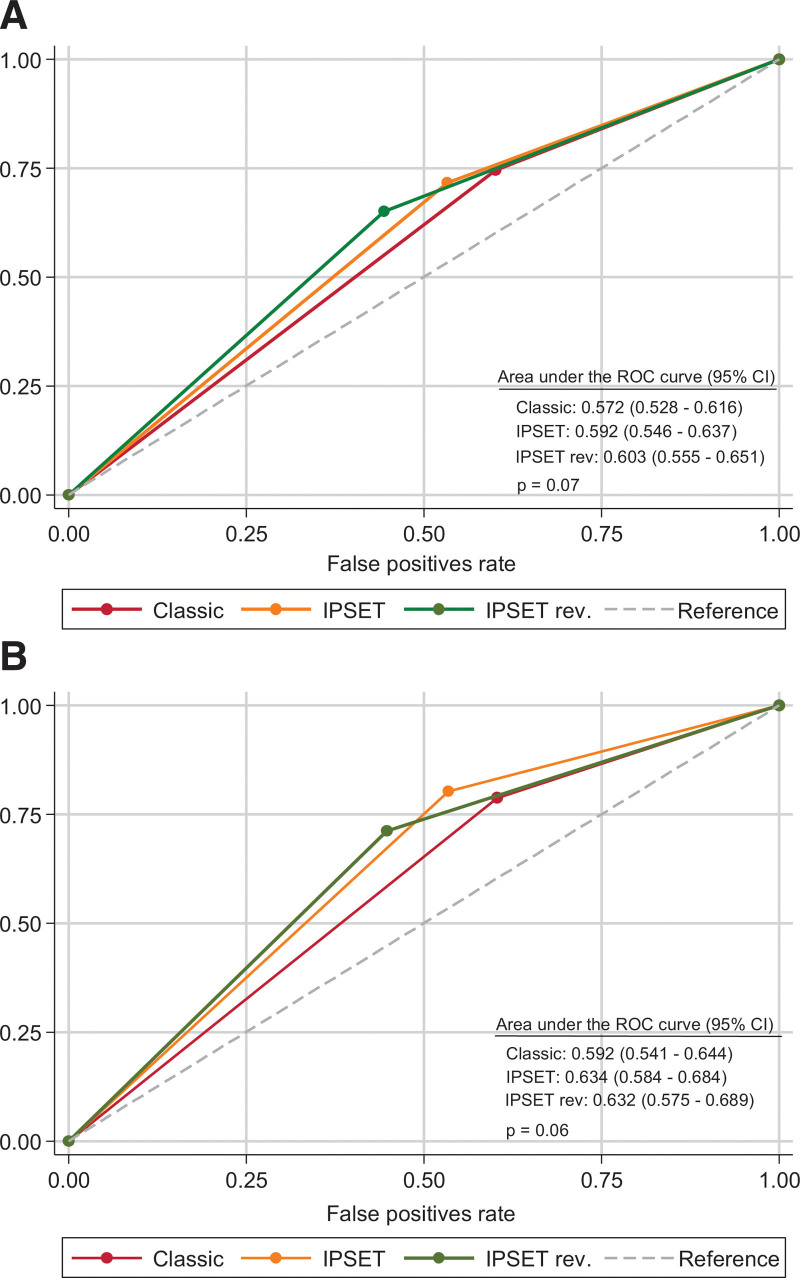
**ROC curves and area under the curve for the IPSET-thrombosis (IPSET), revised IPSET-thrombosis (IPSET-Rev) and the 2-tier classic prognostic model (classic) are showed for the prediction of total thrombosis (A) and arterial thrombosis (B).** The original risk categories of IPSET-thrombosis and revised IPSET-thrombosis have been collapsed into 2strata: high-risk and non-high risk (see text). IPSET-thrombosis = International Prognostic Score of Thrombosis in Essential Thrombocythemia; ROC = receiver operating characteristic.

We then compared the predictive accuracy of the 3 prognostic models by measuring the area under the receiver operating characteristic (ROC) curve. Because the risk of thrombosis did not differ among the low-risk categories in each model, both the IPSET-thrombosis and the revised IPSET-thrombosis were collapsed into only 2 strata: high-risk and non-high-risk of thrombosis. As shown in Figure 3, areas under the ROC curves were not significantly different from each other, neither for the prediction of all thrombotic events nor for arterial ones.

### Dynamic application of revised IPSET-thrombosis model and effect of therapy

The incidence rate of thrombosis was 0.72%, 0.73%, 0.78%, and 1.96% per year in the very low-, low-, intermediate-, and high-risk categories, respectively. To identify groups of patients that might benefit from avoiding cytoreductive therapy, we compared the incidence rates of thrombosis between periods on and off cytoreduction. Since cytoreduction was discontinuous in some patients, the same individual may have contributed to both groups (on and off) at different times during their follow-up. Incidence rates of thrombosis were consistently higher during periods on cytoreduction, although without attaining statistical significance (Table 5). Patients allocated to intermediate risk of thrombosis (aged >60 years, no history of thrombosis, and *JAK2*-negative) were mostly managed with cytoreduction with no thrombotic events registered during periods of time free of cytoreduction (Table 5).

**Table 5:** Incidence of Thrombosis According to the Exposure to Cytoreductive Therapy in 1366 ET Patients Stratified by the Revised IPSET-thrombosis at Diagnosis or During Follow-up

	Cytoreduction			No Cytoreduction				
Revised IPSET-thrombosis Risk Group	Person-years	No. of Events	Incidence Rate (Per 100 p-years)	Person-years	No. of Events	Incidence rate (Per 100 p-years)	Incidence-Rate Ratio (95% CI)	*P*-value
Very low, n = 211 (age <60 y, JAK2-, no history of thrombosis)	589	7	1.2	936	4	0.4	2.8 (0.7-13.0)	0.10
Low, n = 319 (age 60 y, JAK2+, no history of thrombosis)	613	7	1.1	1575	9	0.6	2.0 (0.6-6.0)	0.18
Intermediate, n = 207 (age >60 y, JAK2-, no history of thrombosis)	1088	10	0.9	198	0	0	–	–
High, n = 629 (age >60 y and JAK2+ OR history of thrombosis)	2940	61	2.1	580	8	1.4	1.5 (0.7-3.6)	0.28

CI = confidence interval; IPSET-thrombosis = International Prognostic Score of Thrombosis in Essential Thrombocythemia.

Dynamic application of the revised IPSET-thrombosis model showed a low thrombosis rate when patients in the very low- and low-risk categories changed, respectively, to intermediate- and high-risk categories after reaching 60 years of age. There were no significant differences in the rate of thrombosis when these patients change to a higher risk category after reaching 60 years of age (Table 6).

**Table 6:** Incidence Rate of Thrombosis in Very Low- and Low-risk Patients According to the Revised IPSET-thrombosis at Diagnosis Before and After Reaching 60 Years of Age

Risk Group	From Diagnosis to 60-y Old			After Turning Older Than 60 y			IRR 95%CI	*P*-value
	Person-years	No. of Events	Incidence Rate (per 100 p-years)	Person-years	No. of Events	Incidence Rate (per 100 p-years)		
Very low, n = 211 (age <60 y, *JAK2*-, no history of thrombosis)	1525	11	0.72	511	5	0.98	0.74 (0.24-2.70)	0.56
Low, n = 319 (age <60 y, *JAK2*+, no history of thrombosis)	2188	16	0.73	719	9	1.25	0.58 (0.24-1.50)	0.21

The incidence rate of thrombosis was calculated as the number of events per patient/years in the very low- and low-risk categories while the patients were <60 years and after turning >60 years without history of prior thrombosis (dynamic intermediate- and dynamic high risk, for very low- and low-risk patients, respectively). The 5 thrombotic events registered in the dynamic intermediate group occurred under cytoreduction with no event occurring during 149 person-years of follow-up off cytoreduction. Eight of the 9 thrombotic events registered in the dynamic high-risk group appeared under cytoreduction (incidence rate 1.4% per year), whereas only 1 event occurred off cytoreduction (incidence rate 0.5% per year).

CI = confidence interval; IPSET-thrombosis = International Prognostic Score of Thrombosis in Essential Thrombocythemia; IRR = incidence rate ratio.

## DISCUSSION

The present study constitutes the first evaluation of the IPSET-thrombosis and revised IPSET-thrombosis scores as predictors of thrombosis in a large cohort of ET patients with prospective follow-up. Both scales proved useful for identifying patients at increased risk of arterial thrombosis but failed to predict venous thrombosis. Moreover, after taking death as a competing risk for thrombosis, our results did not reproduce the discriminatory power of the original studies because only 2 well-characterized prognostic risk groups were identified. These results highlight the need to improve thrombotic risk stratification in ET and to develop separate models for venous and arterial thrombosis.

Primary prevention of thrombosis in the general population is based on the estimation of cardiovascular risk at 10 years with most systems estimating the 10-year probability of fatal/nonfatal arterial events, including coronary artery disease, stroke, and/or peripheral artery disease.^11,12^ When applying the revised IPSET-thrombosis in our cohort of patients, the 10-year probability of arterial thrombosis was below 5% in very low-, low-, and intermediate-risk groups. According to the 2019 American College of Cardiology/American Heart Association (ACC/AHA) guidelines on the Primary Prevention of Cardiovascular Disease, patients below the 5% threshold at 10 years are considered at low risk of cardiovascular events and do not need therapeutical intervention.^13^ This finding suggests that ET is not an enhancer for arterial thrombosis in young patients with *JAK2*V617F-mutated ET or in *JAK2*V617F-negative patients of any age. If the estimation of cardiovascular risk by validated scales such as SCORE2 or Framingham might improve the ability to identify patients at higher risk of arterial thrombosis remains to be explored. In the meanwhile, IPSET scores and classic risk stratification are useful tools for selecting patients at high risk of arterial thrombosis candidates to cytoreductive therapy.

The 13% 10-year probability of arterial thrombosis in the high-risk strata of the IPSET-thrombosis scales makes this category similar to the intermediate cardiovascular risk category defined by the ACC/AHA guidelines.^13^ Of note, the majority of IPSET-thrombosis high-risk patients were under cytoreductive plus antiplatelet/anticoagulant treatment at the time of thrombosis, which denotes the need for additional measures to improve thrombosis prevention. First, the high platelet count found in some patients at the time of thrombosis (data not shown) suggests that poor adherence to treatment or inadequate intensity may have lied behind the vascular event in these patients. However, the possible relationship between the normalization of the platelet count and the risk of thrombosis is a matter of debate given the contradictory reported data.^14,15^ Second, following the ACC/AHA guidelines, high-risk IPSET-thrombosis patients might be candidates for moderate-intensity lipid-lowering therapy especially if other well-established enhancers such as a family history of arteriosclerotic cardiovascular disease, persistent low-density lipoprotein cholesterol >160 mg/dL, or renal chronic disease are present.^13^

The annual incidence of VTEs among individuals in their 40s and 50s ranged from 0.08 to 0.2 per 100 population.^16,17^ We observed a 10-year VTE risk of 3.4% and 3.7% in very low- and low-risk patients, respectively, which seems clearly higher than the reported in the age-matched general population.^16,17^ Patients with intermediate and high-risk IPSET-thrombosis scales showed a 5%–6% 10-year probability of venous thrombosis, which is in the range of figures observed in the general population >70 years.^18,19^ Our data indicate that, in comparison with the general population, there is an increased burden of venous thrombosis in ET, more pronounced in young patients. These results are in agreement with a Swedish population-based study in which young MPN patients showed a marked increase of venous thrombotic risk, especially in the first year after diagnosis.^20^ Furthermore, because current scores are inadequate for predicting venous events, newer approaches aimed at stratifying the risk of venous thrombosis in ET are needed, especially for young patients.

An unresolved issue is whether cytoreduction should be omitted in patients over 60 years of age included in the intermediate risk category of the revised IPSET-thrombosis (*JAK2*-negative and no history of thrombosis).^8^ These patients accounted for 15% of our series and faced a risk of thrombosis similar to those in the low- and very low-risk categories, even in the minority not treated with cytoreductive agents. Nevertheless, the nonexperimental design of our study and the relatively low number of patients in the intermediate-risk group managed without cytoreduction precludes drawing any firm conclusion.

Another controversial aspect in the management of ET is whether cytoreduction should be started when a patient progresses on the risk scale for reaching 60 years of age. In our series, in untreated very low-risk patients who progressed to intermediate risk due to age, no thrombotic events were documented, whereas in low-risk patients who transitioned to high risk, only 1 event was recorded while they remained without cytoreduction. However, these data must be interpreted with caution because the follow-up period of these patients is short.

Limitations of the present study include its observational nature, which limits the ability to draw conclusions about therapeutical interventions. In fact, patients treated with cytoreduction showed higher rates of thrombosis than untreated patients in the same risk category, probably illustrating a biased selection by indication. It is worth noting that such a bias can occur when the treating physician has perceived an increased risk for thrombosis not captured by the IPSET-thrombosis scores. In addition, some methodological differences with the seminal studies should be highlighted. We calculated the cumulative incidence of thrombosis in the frame of death as a competing risk instead of recurring to the thrombosis-free survival approach used in the original studies and one real world retrospective study.^4,5,21^ We believe that our approach provides a more clinically useful estimation of the thrombotic risk because both IPSET-thrombosis models are also strong predictors of any-cause mortality.^4,5,9,10^ Finally, we assessed the ability of the IPSET-thrombosis models to predict total thrombosis, arterial thrombosis, and venous thrombosis. Although a connection between arterial and venous thrombosis has been largely proposed, most recent data does not support a clear link between atherosclerosis and venous thrombosis.^22–24^

In conclusion, this prospective study shows that high-risk IPSET categories are useful to identify patients at increased risk of arterial thrombosis whereas failed to predict venous thrombosis. No differences were observed among the other risk categories to predict thrombosis.

## ACKNOWLEDGMENTS

We are indebted to all members of GEMFIN participating in the Spanish Registry of Essential thrombocythemia. We thank MFAR staff for their technical assistance. MPN Spanish Group (GEMFIN) members: Eva María Feijoo, Amparo Cáceres Sansaloni, María Dolores Carrera Merino, Marta Garrote Ordeig, Ana Triguero, Alicia Palomino Mosquera, Raúl Pérez López, Clara Martínez Valverde, Marta García Pintos, Marina Gordillo Martín, Cristina Martínez Bilbao, Carmen García Hernández, Raquel Pla García, Beatriz González Mena, Adriana Simiele Narvarte, José María Guerra, Armando Luaña Galán, Blanca Xicoy Cirici, Anabel Regadera González, Álvaro Díaz González, Rolando Vallansot, Marta Santaliestra, María Antonia Durán Pastor, Marta Hidalgo, José María Raya Sánchez, Cristina Sierra Aisa, M. Teresa Cobo, Juan Manuel Alonso Domínguez, Mercedes Gasior, Miguel Ángel Cortés Vázquez, Alicia Senín.

## AUTHOR CONTRIBUTIONS

AAL designed the study, collected the data, performed the statistical analysis, analyzed and interpreted the results, and wrote the article. AP performed the statistical analysis, analyzed and interpreted the results, and wrote the article. FFM, EAR, and JCHB collected the data, interpreted the results, and wrote the article. BC, PV, SN, GCN, SC, MPE, MTGC, RPL, EM, AM, IPG, AA, MIMV, LCF, JMG, GCT, LF, IM, VGG, and EM collected the data, interpreted the results, and approved the final version.

## DISCLOSURES

The authors have no conflicts of interest to disclose.

## SOURCES OF FUNDING

The Spanish registry of Essential thrombocythemia is financed with GEMFIN’s own funds without direct collaboration from any pharmaceutical company. This work was supported by PI21/00231, PI21/00347, and PI21/00538 from the Instituto de Salud Carlos III (ISCIII), through the Plan Estatal de Investigación Científica y Técnica y de Innovación.

## Supplementary Material


