# Supplementary material for: Application of IPSET-thrombosis in 1366 Patients Prospectively Followed From the Spanish Registry of Essential Thrombocythemia
Source: Hemasphere. 2023 Jul 18;7(8):e936. doi: 10.1097/HS9.0000000000000936 (PMC10356121; doi:10.1097/HS9.0000000000000936)
Supplement: Supplementary file 1 [file hs9-7-e936-s001.pdf]

SUPPLEMENTAL FIGURE 1

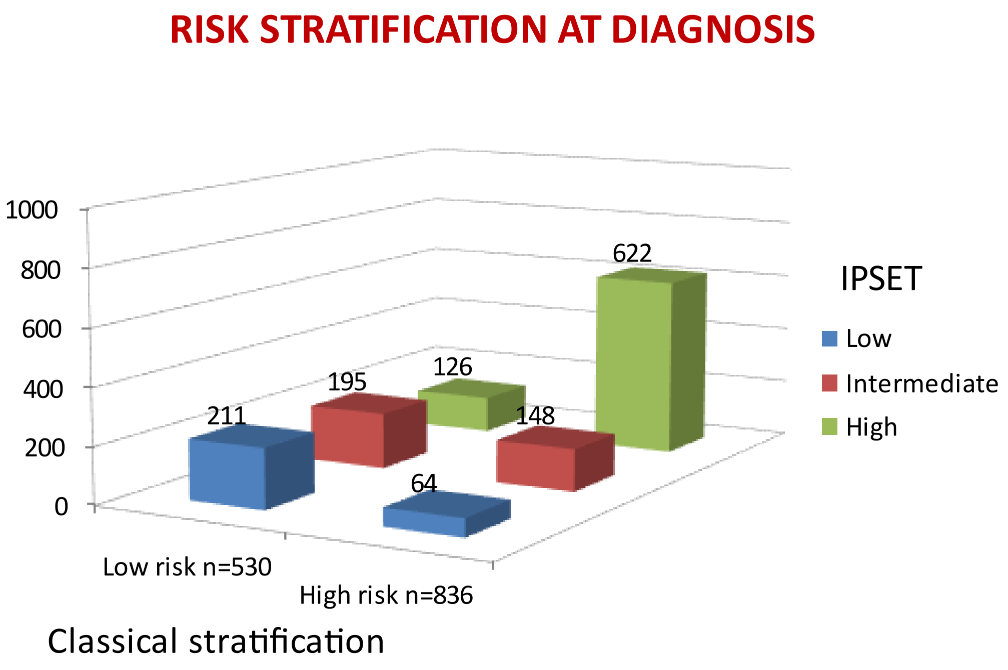

SUPPLEMENTAL FIGURE 2

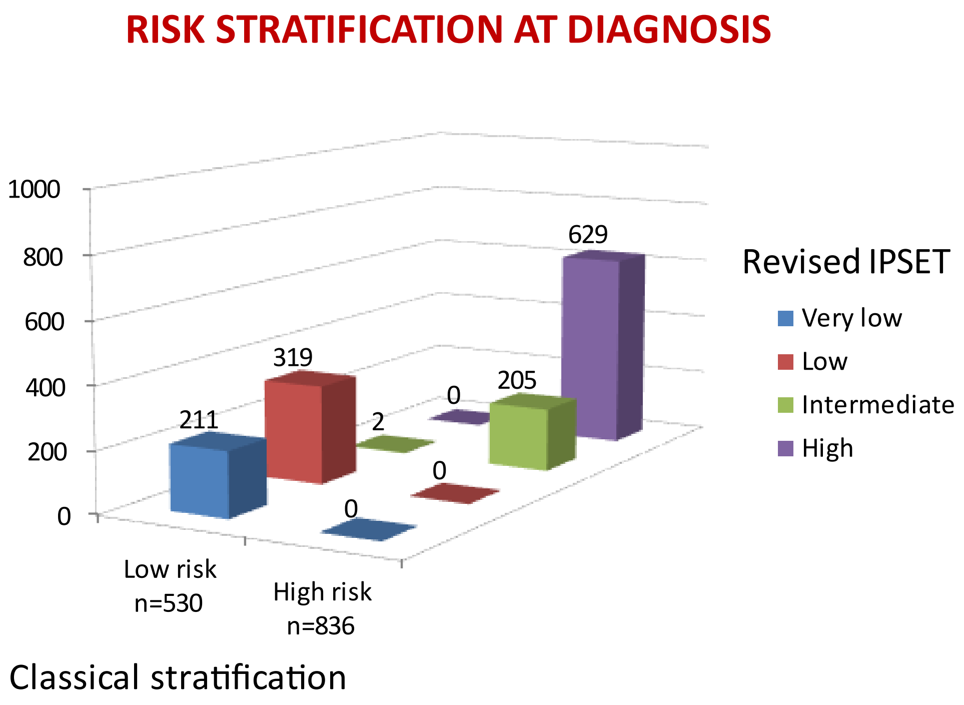

SUPPLEMENTAL FIGURE 3

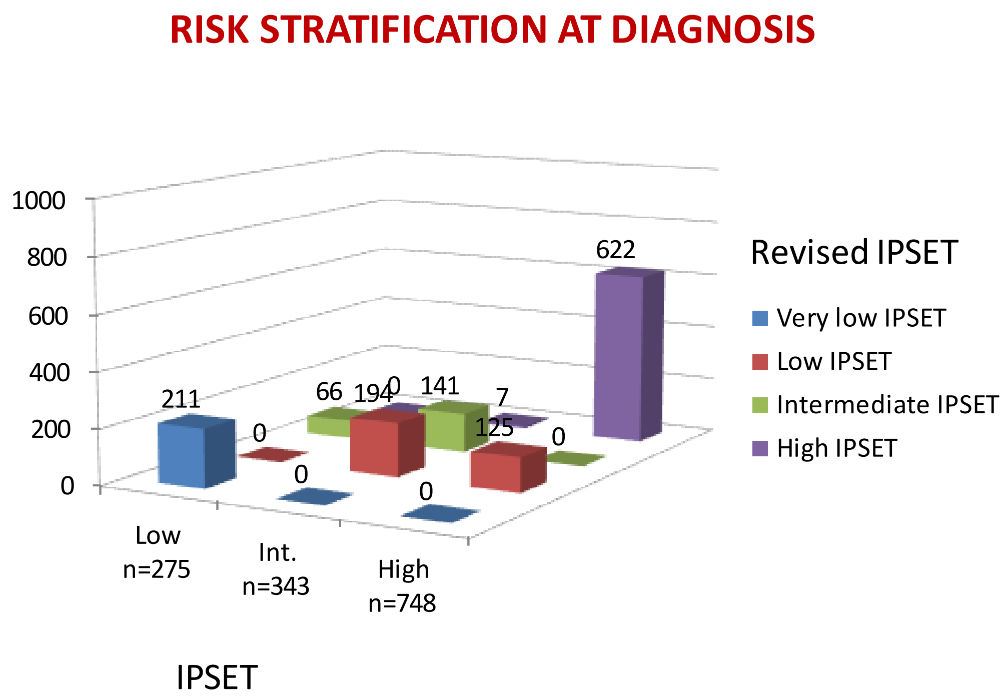

SUPPLEMENTAL FIGURE 4

Survival according to the risk categories of IPSET-thrombosis, revised IPSET-thrombosis and the classic 2-tier model for the stratification of thrombotic risk in ET. The table summarizes the hazard ratios (HR) and their 95% confidence intervals (CI) as compared with the reference risk category.

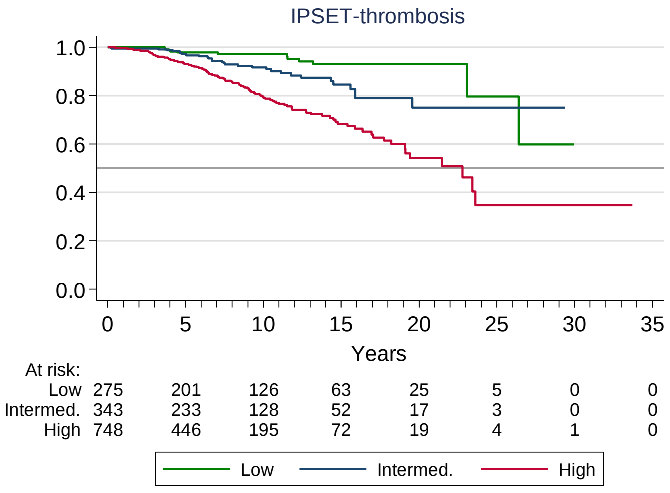

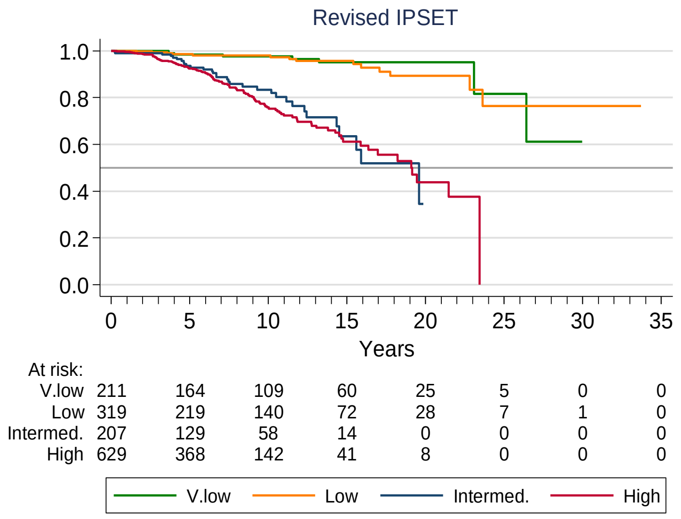

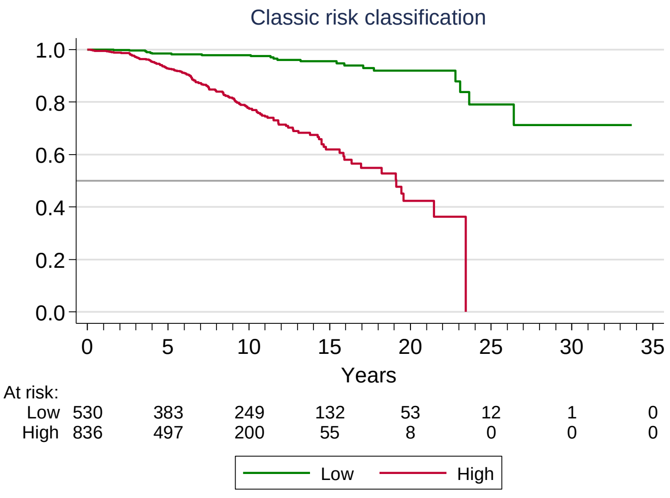

Risk category	HR (95% CI)	p
**IPSET** (reference category: low-risk)		
Intermed.	2.4 (1.2 – 4.7)	0.01
High	5.6 (3.1 – 10)	< 0.001
**Revised IPSET** (refeference category: very low risk)		
Low	1.2 (0.5 – 3.0)	0.6
Intermed.	8.9 (4.0 – 18)	< 0.001
High	10.4 (5.0 – 22)	< 0.001
**Classic** (reference category: low-risk)		
High	8.8 (5.4 – 14)	< 0.001
