# Supplementary material for: Application of IPSET-thrombosis in 1366 Patients Prospectively Followed From the Spanish Registry of Essential Thrombocythemia
Source: Hemasphere. 2023 Jul 18;7(8):e936. doi: 10.1097/HS9.0000000000000936 (PMC10356121; doi:10.1097/HS9.0000000000000936)
Supplement: Supplementary file 2 [file hs9-7-e936-s002.docx]

| **Supplemental table 1. Causes o death in 1366 patients prospectively followed rom the Spanish Registry o Essential Thrombocythemia** | |
| --- | --- |
| Cardiovascular | 31 |
| Disease progression | 14 |
| Infection | 47 |
| Second primary cancer | 27 |
| Neurodegenerative disease | 4 |
| Other | 8 |
| Not reported | 32 |
| Total | 163 |

Results are expressed as number of patients
