# Supplementary material for: Application of IPSET-thrombosis in 1366 Patients Prospectively Followed From the Spanish Registry of Essential Thrombocythemia
Source: Hemasphere. 2023 Jul 18;7(8):e936. doi: 10.1097/HS9.0000000000000936 (PMC10356121; doi:10.1097/HS9.0000000000000936)
Supplement: Supplementary file 3 [file hs9-7-e936-s003.docx]

| **Supplemental table 2. Number and type of thrombotic events in 1366 patients with prospective follow-up from the Spanish Registry of Essential Thrombocythemia** | | | |
| --- | --- | --- | --- |
|  | Prior to diagnosis* | At Diagnosis | During Follow-up** |
| **Arterial thrombosis**  CAD  Stroke/TIA  PAD  Other | **152**  50  82  15  5 | **49**  17  25  7  - | **66**  18  41  6  1 |
| **Venous thrombosis**  Superficial thrombophlebitis  DVT/PE  SVT  Other | **58**  36  8  8  6 | **24**  3  9  11  1 | **40**  3  27  8  2 |
| **Total** | **210** | **73** | **106** |

*Some patients contributed with more than one event **First thrombotic event after diagnosis
